# What patients want from access to UK general practice: systematic review

**DOI:** 10.3399/BJGP.2024.0582

**Published:** 2025-07-01

**Authors:** Helen Atherton, Helen Leach, Rob Mortell, Joanne Parsons

**Affiliations:** 1 Primary Care Research Centre, University of Southampton, Aldermoor Health Centre, Southampton, UK; 2 Warwick Medical School, Coventry, UK; 3 Yeovil District Hospital, Yeovil, UK; 4 Centre for Evidence and Implementation Science, University of Birmingham, Birmingham, UK

**Keywords:** access to primary care, general practice, health services accessibility, patient experience, primary health care

## Abstract

**Background:**

Access to general practice is a topical concern, with rising numbers of consultations and decreasing numbers of GPs placing strain on the service. Patient satisfaction with general practice has seen a reduction in the UK. While patient experience with general practice is well understood, there is a need to understand what patients say they want from access to general practice.

**Aim:**

To examine what patients want from access to contemporary general practice in the UK.

**Design and setting:**

This was a systematic review set in UK general practice.

**Method:**

Studies were included that reported patient wants in relation to access to general practice in the UK since 2010. All empirical study designs were included, both quantitative and qualitative. The mixed-methods appraisal tool was used to assess study quality for contextual purposes. Narrative synthesis was applied to the included studies, with results presented using tables and text.

**Results:**

In total, 33 studies were included. The review showed that patients wanted information about how to access the general practice, choice of clinician, choice of healthcare professional type, and choice of consultation mode. Patients wanted a nearby practice, with clean waiting rooms, easy appointment booking using simple systems and with short waiting times, and to be kept informed about the process.

**Conclusion:**

The factors that patients want should be taken into consideration when changing or developing approaches to access. Future evaluations of care, and research, should explicitly consider what patients want from access in general practice.

## Introduction

Access to general practice is a topical issue, a point of focus for politicians, and subject to negative newspaper headlines that emphasise the perceived inaccessibility of appointments with a GP.^1^ In the UK, recorded patient satisfaction with general practice has decreased in recent years^2^ and consultation numbers have risen to 34.3 million in 2023 from 30.8 million in 2019.^3^ At the same time, the number of full-time equivalent GPs has reduced,^3^ leading to reduced capacity to deliver appointments. This has affected several countries around the world.^4–7^

Attempts to manage demand for appointments in general practice have led to initiatives ranging from allied health professionals consulting with patients^8^ to patient education focused on self-care.^9^ Recently pharmacies were given approvals to directly prescribe medication for minor illnesses.^10^ Alongside this, there have been changes in recent years to how patients access and consult in general practice, with increased reliance on telephone or online triage approaches,^11,12^ and use of remote consultation^13^ with this change accelerated by the COVID-19 pandemic.

Given these changes, and negative media perceptions about general practice,^14^ it is timely to examine what patients actually report they want from access to general practice. This study, therefore, sought to examine what UK patients report they want from general practice, via systematic review. This allowed the authors to summarise what is important to patients in accessing modern UK general practice.

## Method

The protocol for this systematic review is published (PROSPERO CRD42021268073) and this review is reported using PRISMA guidelines.^15^

### Inclusion and exclusion criteria

Studies were included that stated patient-reported wants concerning access to contemporary general practice in the UK.

**How this fits in**

Widely accepted, as perpetuated by the media, is that patients are unhappy with access to general practice and desire faster access to a GP. This review sought to summarise the research evidence about reported patient wants from access to general practice. Patients wanted to easily make an appointment in a timely fashion, to have a positive relationship with the practice, to see a specific clinician, and choose consultation modality according to individual circumstances. Communication and being kept informed about access throughout the process of making and having an appointment was something patients wanted, and this could be addressed by general practice.

Access to general practice was defined as being one or more of the following domains as reported in Simpson *et al*,^16^ chosen for the pragmatic nature of the categories that allowed grouping of the data rather than inferring interpretation:

- physical access: for example, the availability of GPs, distance and transport, design of premises, the availability of home visits;
- timely access: for example, the availability of appointments and the satisfaction of patients with opening hours; the provision of out-of-hours care and waiting times; and
- choice: for example, the choice of practice and choice of professional.

A want was defined as a patient-stated desire for something (for example, for access to be convenient).

Studies were included:

- where patient-stated wants were in relation to accessing UK general practice for an appointment;
- that used empirical study designs;
- that had been published since 2010;
- that were published in the English language;
- where patient-reported data could be extracted from studies that examined both patient and staff perspectives; and
- where patient-related data on access could be extracted from studies that examined factors beyond access.

Studies were excluded where:

- patient-stated wants in relation to access were examined for a specific condition or patient group only;
- satisfaction and/or experience was measured and did not elicit want;
- healthcare professionals reported their views or that of patients; and
- patients were using general practice outside of the UK, using private GP services, or out-of-hours services.

The date 2010, when there was a change in UK Government after 13 years, was chosen as the start date for the search to reflect political and managerial changes in the NHS after that time.

### Search strategy

All the main medical databases were searched. The search strategy was informed by literature^17–19^ and supported by an academic support librarian. For Medline strategy and databases search see Supplementary Appendix S1.

Searches included all records from January 2010 and were run on 16 May 2024.

### Screening

Covidence software was used to enable screening. Titles and abstracts were screened by two authors independently. Full texts were retrieved for studies meeting the inclusion criteria and screened. Reasons were recorded for studies excluded at full-text stage. Studies meeting the inclusion criteria at the full-text stage were included in the review. Reference lists of all included studies were screened. Any discrepancies during screening were resolved by a third author.

### Outcome measures

Outcomes of interest were patient-expressed wants in relation to access, taking any form.

### Data extraction

Data were extracted by two authors independently. Information was extracted relating to study design, setting, participants, and patient wants from access to general practice.

### Quality assessment

The Mixed Methods Appraisal Tool (MMAT) version 2018^20^ was used to assess quality of included studies. This tool is appropriate for conducting quality appraisal of quantitative, qualitative, and mixed-methods studies in systematic mixed-study reviews. It provides a thorough quality assessment by assessing each study according to the study design and provides a validated way to compare different study designs in relation to quality,^20^ without biasing the process for or against any given study design. Studies are given an overall quality rating based on five criteria relevant to the study design: high quality (meet four or five criteria), moderate quality (meet three criteria), or low quality (meet none to two criteria). The results of the assessment for the purposes of context and interpretation are reported.

### Data analysis

Owing to the heterogeneity of the study data, a narrative synthesis approach was used, presenting the findings using tables and text,^21^ looking at themes across the data. Narrative synthesis enables studies with different designs to be analysed in a systematic way considering the similarities and differences between the studies.

## Results

**Figure 1.**
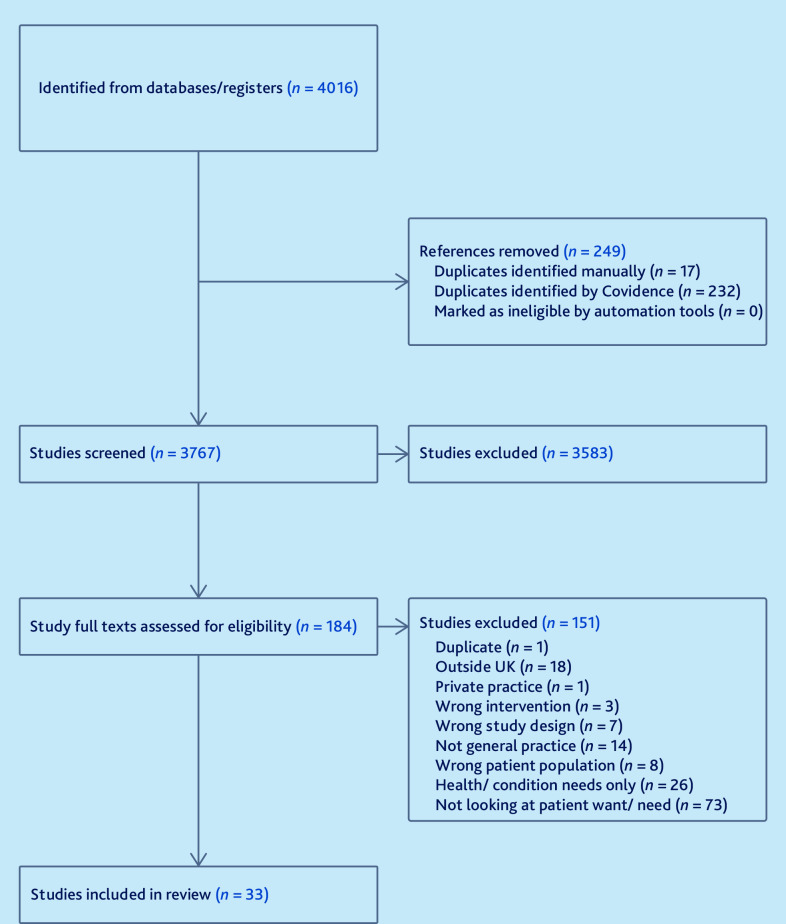
PRISMA flow diagram

Searches resulted in 4016 results. There were 33 studies included in this review.^22–57^ See Figure 1 for the PRISMA flow diagram.

There were 15 studies that used a qualitative design, eight that used a mixed-methods design, and 10 that used a quantitative design (see Supplementary Table S1). Of the quantitative studies, five conducted secondary analysis of the English GP Patient Survey.^22–26^ Three studies were set exclusively in Scotland,^27–29^ two in England and Scotland^30–32^ and with three described as being set in the UK.^34,35,37^ The remaining studies were set in England. Six studies included data collected from March 2020 onwards.^12,29,37–40^ The full characteristics of each study are outlined in Supplementary Table S1.

### Quality assessment

Three included studies^28,41,42^ were rated of low quality, two of moderate quality,^32,38^ and the remaining 28 were of high quality (Supplementary Table S2). Of the two studies of moderate quality, both were quantitative studies, with one lacking detail on the sample and about non-responders, and the other with a non-representative sample and no information on non-responders. All three studies rated of low quality were mixed-methods studies where there was no formal integration between methodological approaches, and lack of discussion about divergences and inconsistencies across studies.

Results are presented in four groups: connection, choice, timely access, and physical access. These are also summarised in Supplementary Table S3.

### Connection

#### Relationship with the practice

Patients expected receptionists to greet them,^43^ and to be friendly and polite, to be treated with respect and not as ‘just another patient’.^28^

#### Communication from the practice

Patients wanted to know how to get evening, night, and weekend appointments.^44^ During the COVID-19 pandemic, patients wanted information about how to access general practice.^38^

Where a practice had healthcare professionals in multiple roles, patients wanted to know about their services, skills, and competencies, and when they should access them.^45–47^

### Choice

#### Preferred clinician and continuity of care

In several studies, patients reported wanting to choose a specific clinician when making an appointment,^22,26,28,43,46–50^ ensuring relational continuity.^46,47,50^ For medication reviews and long-term conditions patients preferred seeing someone they were familiar with.^28,51^

Sometimes patients were happy to see any doctor,^43^ and when using an online consultation patients mostly preferred speed of response over continuity of care,^12^ although some patients reported wanting a response from a specific GP.^12,42^ Wanting to see a specific doctor was more important when requesting non-urgent appointments (odds ratio 1.40, 95% confidence interval [CI] = 1.39 to 1.42).^22^

#### Skill mix

Those in ethnic minority groups and those not confident in managing their own health were more likely to want to see or speak to a GP over other healthcare professional types, and older patients were less likely to want to see or speak to a GP rather than a nurse.^25^

Where a patients condition was worsening 69.5% (73/105) of patients reported preferring to consult a GP than a pharmacist^32^ and 42.7% (44/103) strongly agreed or agreed that they would prefer to consult with a GP rather than a pharmacist prescriber given the choice.^32^

Patients wanted the choice to consult with a general practice-based pharmacist when making an appointment^45^ and in a different study expressed a ‘strong willingness’ to see the pharmacist again over a GP.^52^ However, 14.3% (11/77) of patients seeing a pharmacist prescriber felt they would still need to consult a GP after their appointment.^41^ Where patients wanted to see or speak to a GP and instead spoke to a nurse, patients reported low confidence and trust scores in the healthcare professional (adjusted mean difference –15.8, 95% CI = –17.6 to –14.0).^25^

#### Consultation modality

Patients wanted to be able to choose consultation modality.^34,35,38^ Patients wanted to use and initiate email consultations,^53^ but for online consulting that involved a form, or for telephone consultations, patients wanted to be able to book a face-to-face consultation instead.^34,38,48,49^ In one study 76.2% (582/763) of patients reported they wanted to use online consultation again instead of booking a face-to-face consultation.^54^ For test results, patients wanted to choose the modality.^55^ Patients wanted to use an online platform flexibly 24/7 rather than when made available by the practice.^12,29^

Some patients felt that new health problems were better suited to face-to-face appointments,^30,31^ and they had a preference for these,^29^ regarding them as the ‘gold standard’^27,35^ that should always be available.^38^ In one study, patients wanted a face-to-face appointment when medication changes were required.^51^

Patients were shown in a principal components analysis to be negative about the prospect of remote consultations continuing after the pandemic.^37^ In another study 10.3% (116/1132) of patients wanted to get rid of online consultation.^42^ Where patients had wanted a face-to-face appointment with a GP but had a telephone appointment with a GP instead, their scores for confidence and trust in the healthcare professional were lower than for a face-to-face appointment (adjusted mean difference –6.47, 95% CI = –7.00 to –5.93).^25^

### Timely access

#### Ease of booking

Patients wanted to be able make an appointment easily.^28,44^ They wanted better organisation of booking systems, for example, fewer options to press and a shorter automated message when calling the practice,^29,38^ a queuing system,^55^ advance notice of when they might receive a telephone callback,^28,33,38,39^ or the ability to drop in to make an appointment.^48,49^ Patients wanted a website that had a clear route to use online services,^42^ and did not want multiple routes.^39^

Asynchronous remote consulting, such as email or online consultation use, was particularly helpful for patients who would prefer to bypass booking an appointment with the receptionist.^30,33,42,53^ Patients wanted to be able to access the GP for anything they deemed serious.^29^ For test result communication, they wanted increased accessibility of GPs for this purpose.^55^

#### Speed of access

Patients preferred short wait times.^29,36,44^ Remote consulting was seen as offering convenience,^48,49,53,54^ which patients wanted, allowing flexibility around working or caring commitments.^30,31,40,52,53^

This convenience was caveated by wanting responses or callbacks to be prompt.^27,42^ For online requests, they wanted to receive acknowledgement of receipt.^12,29^ Patients also wanted to be seen on time^28^ and to access longer consultations.^29^ Patients valued the ability to book an appointment at a time or date most suited for them and where needed, for same-day/urgent appointments^48,49^ as well as future access.^28^

Even when patients were satisfied with appointment times there was still demand for extended access outside traditional office hours^23,24,45^ and for working patients weekday lunchtime access was favourable.^36^ In another study, of the 19.1% (168 407/881 183) responders who reported their practice was not open at a convenient time, 76.1% (670 580/881 183) felt that Saturday or Sunday access would make it easier to have an appointment.^23^

### Physical access

#### Getting to the practice and practice premises

Patients wanted practices to be within a neighbourhood rather than outside of it,^36^ or wanted it close to their home.^44^ Having first-contact physiotherapists within their community, as opposed to in less local hospital settings, was preferred.^56^ Patients reported the desire for clean, calm, and well-managed waiting rooms.^43^

#### Use of remote consultations

Patients using remote consultations reported not wanting to travel to the practice^30,31,42,48,49^ with reasons including mobility or health issues,^30,31,40,53,54^ not being able to take time off work or pay travel costs,^27,54^ or having childcare to arrange.^40,42^ Asynchronous access was desirable for patients with communication difficulties such as hearing disabilities^30,31^ and for those with work commitments.^42^ Patients who wanted privacy could not necessarily achieve this with a telephone consultation, potentially compromising confidentiality.^49,57^ Video allowed for visual cues that patients wanted but were not available in telephone consultations.^27^

## Discussion

### Summary

Data on patient wants from access to general practice were limited, with research mainly focused on experience and satisfaction with access. This review shows that patients wanted a positive relationship with the practice, wanting information about where to seek care. When it came to healthcare professional and consultation modality, patients wanted choice, ideally wanting to see a GP or a specific clinician. Patients wanted the ability to easily make an appointment, with a short wait time. They wanted simple booking systems, which kept patients informed and were easily accessed. Patients wanted the general practice to be nearby, with clean waiting rooms. If patients wanted a remote consultation, it was to avoid travelling to the practice.

### Strengths and limitations

This review focused on patient wants, taking a novel perspective on how patients view access. Research published since 2010 only was assessed with a risk that important studies from before then were missed. The review examined studies from the UK, which limits the transferability of the findings. Access to a general practice appointment was examined, but there are other aspects of access such as access to information that promotes self-care that were not included in this review despite forming part of the wider access landscape. The review focused on studies that examine the general practice population as a whole and studies that examined specific patient groups were not examined. This means the findings do not include data that indicate how what patients want from access to general practice varies by condition or other potentially relevant demographic characteristics. Excluding these studies allowed the authors to take a high-level view on what patients want but future studies could focus on the nuance between what different patient groups with particular conditions or characteristics want from access to general practice. The review did not include grey literature and may have missed patient voices in research by advocacy organisations such as Healthwatch. This review has only presented the wants of patients whose views are documented in research, and this may exclude patients with the greatest healthcare need as they are less likely to take part in research. The included studies were mostly rated as being of high quality, and data from all studies were included regardless of quality. Those of low quality were mixed-methods studies that scored poorly on integration of data between methods. This may reflect the challenges of using a mixed-methods approach to assess a complex area such as access.

### Comparison with existing literature

A study published in 2003 used secondary analysis of data from a large-scale survey in general practice to examine patients’ views on access and continuity. They identified that satisfactory standards of access included next-day appointments and a 6–10-minute wait for consultations to begin, as well as seeing the same GP. Over 20 years later, the current study has presented findings that indicate these same things are still important to patients.

In the Additional Roles Reimbursement Scheme (ARRS) in the UK, general practices are funded to employ a varied range of healthcare professionals to increase capacity for appointments. A recent study found that adding an ARRS role to general practice was associated with a one-percentage-point increase in the proportion of patients able to make an appointment.^58^ The current findings indicate a lack of confidence from patients when seeing a professional other than a GP, which is not necessarily being considered when examining the increased capacity provided by these roles.

### Implications for research and practice

More patients than ever are seeking consultation with a GP^3^ and this contact is managed via access systems that filter patients according to clinical need,^59^ with demand often deemed inappropriate, excessive, or unwarranted by both general practice staff and patients themselves.^60,61^ Understanding and addressing patient wants may be an important part of understanding why demand has increased and appears to be excessive or unwarranted, and could feed into changes in how access to general practice is delivered.

There is scope for future research to consider in more depth what patients want, and to encourage co-production with patients of solutions to access problems faced by general practice.

The General Practice Patient Survey conducted by NHS England collects data on patient satisfaction with general practice and has led to several research studies.^62,63^ This review only included those studies where questions relating to what patients wanted were addressed and there are few of these. This survey could feasibly be modified in future to collect data on what patients want from their general practice.

Collecting high-quality evidence on what patients want from general practice, and considering how to provide it, may hold potential to address the access crisis.

## Data

All studies included in this review are published and publicly available.
